# 45852 Parenting stress predicts fast food intake in an urban community sample of overweight parents of toddlers

**DOI:** 10.1017/cts.2021.494

**Published:** 2021-03-30

**Authors:** Tara Bautista, Nia Fogelman, Schan Lartigue, Wendy K. Silverman, Ania M. Jastreboff, Rajita Sinha

**Affiliations:** 1 Yale School of Medicine; 2 New York Medical College

## Abstract

ABSTRACT IMPACT: The findings suggest that targeting parenting stress in combination with psychoeducation on nutrition and physical activity may have positive effects in improving healthy food choices such as reduction of fast food intake, which may in turn impact the health of toddlers and their families. OBJECTIVES/GOALS: Parent stress is associated with a myriad of unhealthy behaviors including overeating, decreased physical activity, which contribute to increased weight. Several programs have aimed to increase education of nutrition, but few have focused on parent stress to improve healthy food intake. The present study assessed parent stress and fast food intake. METHODS/STUDY POPULATION: Parents who have obesity and had a toddler in the age group of 2-5 years were enrolled for a preventive intervention study to assess the effect of a parent-based intervention to improve family health choices and reduce childhood obesity risk. The sample included 105 participants, mean age 34.80 (6.27) years old, mean body mass index (BMI) 35.51 kg/m2, 39.0% Non-Hispanic White, 20.0% Non-Hispanic Black, 22.9% multiracial, 12.4% Hispanic, and 5.7% other. Stress was assessed using the Perceived Stress Scale (PSS) to assess overall general stress and the Parenting Stress Index (PSI) to assess parent-specific stress. Chaos in the home and fast food intake were also assessed using self-report surveys. RESULTS/ANTICIPATED RESULTS: Preliminary results are based on available data as of October 2020, data collection and recruitment are still in progress. There was a significant correlation between fast food intake with PSS (r=.18, p=.04), chaos (r=.24, p=.02), and PSI (r=.25, p=.01). Using a hierarchical regression model, we entered home chaos in the first block which explained a significant amount of the variability (R2= .06, p=.04). PSS was entered in the second block, which was not significant (R2 change=.01, p=.50), and in the final block PSI was entered and was significant (R2 change=.13, p <.01). DISCUSSION/SIGNIFICANCE OF FINDINGS: The data indicate that parenting stress uniquely predicts fast food intake above and beyond what could be explained by home chaos and general perceived stress. Future analyses will assess a parent-based intervention targeting stress reduction to improve weight and health for the parent and their toddlers in order to reduce childhood obesity risk.

